# Metabolic adaptation is not a major barrier to weight-loss maintenance

**DOI:** 10.1093/ajcn/nqaa086

**Published:** 2020-05-09

**Authors:** Catia Martins, Barbara A Gower, James O Hill, Gary R Hunter

**Affiliations:** Obesity Research Group, Department of Clinical and Molecular Medicine, Faculty of Medicine and Health Sciences, Norwegian University of Science and Technology (NTNU), Trondheim, Norway; Centre for Obesity and Innovation (ObeCe), Clinic of Surgery, St. Olav University Hospital, Trondheim, Norway; Department of Nutrition Sciences, University of Alabama at Birmingham, Birmingham, AL, USA; Department of Nutrition Sciences, University of Alabama at Birmingham, Birmingham, AL, USA; Department of Nutrition Sciences, University of Alabama at Birmingham, Birmingham, AL, USA; Department of Nutrition Sciences, University of Alabama at Birmingham, Birmingham, AL, USA

**Keywords:** metabolic adaptation, adaptive thermogenesis, weight regain, energy expenditure

## Abstract

**Background:**

The existence of metabolic adaptation, at the level of resting metabolic rate (RMR), remains highly controversial, likely due to lack of standardization of participants’ energy balance. Moreover, its role as a driver of relapse remains unproven.

**Objective:**

The main aim was to determine if metabolic adaptation at the level of RMR was present after weight loss and at 1- and 2-y follow-up, with measurements taken under condition of weight stability. A secondary aim was to investigate race differences in metabolic adaptation after weight loss and if this phenomenon was associated with weight regain.

**Methods:**

A total of 171 overweight women [BMI (kg/m^2^): 28.3 ± 1.3; age: 35.2 ± 6.3 y; 88 whites and 83 blacks] enrolled in a weight-loss program to achieve a BMI <25, and were followed for 2 y. Body weight and composition (4-compartment model) and RMR (indirect calorimetry) were measured after 4 wk of weight stability at baseline, after weight loss and at 1 and 2 y. Metabolic adaptation was defined as a significantly lower measured compared with predicted RMR (from own regression model).

**Results:**

Participants lost, on average, 12 ± 2.6 kg and regained 52% ± 38% and 89% ± 54% of their initial weight lost at 1 and 2 y follow-up, respectively. Metabolic adaptation was found after weight loss (−54 ± 105 kcal/d; *P* < 0.001), with no difference between races and was positively correlated with fat-mass loss, but not with weight regain, overall. In a subset of women (*n* = 46) with data at all time points, metabolic adaptation was present after weight loss, but not at 1- or 2-y follow-up (−43 ± 119, *P* = 0.019; −18 ± 134, *P* = 0.380; and − 19 ± 166, *P* = 0.438 kcal/day respectively).

**Conclusions:**

In overweight women, metabolic adaptation at the level of RMR is minimal when measurements are taken under conditions of weight stability and does not predict weight regain up to 2 years follow-up.

The JULIET study is registered at https://clinicaltrials.gov/ct2/show/NCT00067873 as NCT00067873.

See corresponding editorial on page 501.

## Introduction

The reduced obese state is associated with a significantly reduced total energy expenditure (TEE), attributable to both a reduction in resting and nonresting energy expenditure (EE) (1). Even though this reduction is likely to be mainly driven by the loss of metabolically active tissue (2–6), some have reported a reduction in TEE and/or its components (resting and nonresting EE) in excess of what would be predicted, given the measured losses in both fat mass (FM) and fat-free mass (FFM) (1, 7–10), a mechanism known as metabolic adaptation or adaptive thermogenesis.

This conservation of energy (or “hibernation mode”), activated in response to weight loss, has been one of the most controversial issues in the obesity field, not only in terms of whether it exists but also its clinical relevance if it exists. It has been suggested to be a potential explanatory mechanism for resistance to weight loss and an important driver of long-term weight regain (relapse) (11–15). Some have argued that the claims around metabolic adaptation are exaggerated (16, 17) and others shown that when weight-stable obesity-reduced individuals are compared with BMI-matched controls (2, 4, 18, 19), or against a prediction equation (20), no evidence of metabolic adaptation at the level of resting metabolic rate (RMR) exists. Even though it has been argued that discrepancies among studies derive from lack of accuracy and precision in the measurement of EE and body composition (11, 12), we hypothesize here that differences among studies derive from inconsistencies related to the status of energy balance (EB) and/or weight stability of the participants when measurements are taken. The requirement of EB status is likely to be important not only for baseline measurements, when regression models are derived from own data, but also subsequent post–weight-loss data, when metabolic adaptation is investigated, as the difference between measured and predicted values.

Obesity rates are higher in blacks than in whites (21), and black women consistently lose less weight in response to lifestyle interventions (22–24). Differences in metabolic adaptation between races could contribute to this and at least 1 study has shown that the reduction in RMR observed with weight loss is larger in blacks than in whites, after adjusting for body-composition changes (25). However, the great majority of the studies on metabolic adaptation have been performed in whites.

Therefore, the primary aim of this analysis was to determine whether metabolic adaptation, at the level of RMR, exists in a population of premenopausal overweight women, with measurements taken in conditions of weight stability (4 wk) at baseline, after weight loss, and also at 1- and 2-y follow-up. Secondary aims were to look at race differences (whites compared with blacks) in metabolic adaptation and to investigate if metabolic adaptation was correlated with weight regain at 1- or 2-y follow up.

## Methods

### Participants

Participants in this analysis were premenopausal overweight women. They were 20–41 y of age, sedentary (no more than 1 time/wk of regular exercise), had normal glucose tolerance (2-h glucose ≤140 mg/dL following 75-g oral dose), family history of overweight/obesity in ≥1 first-degree relative, and no use of medications that affect body composition or metabolism. All women were nonsmokers and reported a regular menstrual cycle. Race (white or black) was self-reported (participants were considered white if they answered that all 4 grandparents were white, and black if they reported all 4 grandparents to be black). The 2 studies included in this retrospective analysis were both approved by the Institutional Review Board for Human Use at the University of Alabama at Birmingham (UAB). All women provided informed consent before participating in the study.

### Study design

Participants included in this retrospective analysis were from 2 different studies [1: energy expenditure in postobese black and white women (ROMEO); 2: exercise training in obesity-prone black and white women (JULIET)], performed at the Department of Nutritional Sciences at UAB, with exactly the same sequence of events (see flowchart, **Supplemental Figure 1**) and the same methodology and both aiming to identify metabolic predictors of weight regain. In the ROMEO study, all participants achieved weight loss with diet alone (single-arm longitudinal study with repeated measurements). In the JULIET study, participants were randomly assigned to 1 of 3 groups: *1*) weight loss with aerobic exercise training 3 times/wk, *2*) weight loss with resistance exercise training 3 times/wk, or *3*) weight loss with diet alone (same diet as in ROMEO). Of note, no significant differences in metabolic adaptation were seen between groups. During weight loss, all participants were provided an 800-kcal diet until reaching a BMI (kg/m^2^) <25. Food was provided (20–22% fat, 20–22% protein, and 56–58% carbohydrate) by the General Clinical Research Center (GCRC) kitchen. During the first follow-up year, participants were encouraged, but not mandated, to attend regular support-group meetings (bimonthly dietary education classes aimed at weight maintenance for the first 6 mo, followed by monthly meetings for months 6–12) and to continue with their exercise program, if applicable. For detailed information about the ROMEO and JULIET studies, see Weinsier et al. (3) and Hunter et al. (26).

Testing was done, after a 4-wk weight-stabilization period (aiming to maintain body weight within a 2.5-kg range), at baseline, after weight loss, and at 1- and 2-y follow up. Testing was done 30 ± 2 d after the end of the weight-loss phase. During the 4-wk weight-stabilization period, participants were weighed 3 times/wk the first 2 wk while consuming their own food and weighed 5 times/wk with food provided by the GCRC the last 2 wk. Variation in body weight during the last 2 wk of the stabilization period, after weight loss, was −1.0 ± 1.4 kg. All testing was conducted in the follicular phase of the menstrual cycle during a 4-d GCRC inpatient stay (to ensure that physical activity and diet were standardized). Testing was done in a fasted state in the morning after spending the night in the GCRC.

### Data collection

The following measurements were conducted at baseline, after weight loss, and at 1- and 2-y follow-up, after a 4-wk weight-stabilization period (at all time points).

#### Body weight and composition

Body composition was determined by using the 4-compartment model (4CM) (27), which includes in the analysis bone mineral content, total body water, and total body density to take into consideration interindividual variations in body density and the fact that black women generally have a greater bone mineral content than do white women (28). The 4CM includes the following density assumptions: 0.9 kg/L for fat, 0.99 kg/L for water, 3.042 kg/L for total mineral (osseous and cellular), and 1.34 kg/L for the unmeasured fraction of the body composed of protein and glycogen. The model is used to calculate the percentage of FM from independent measures of total body density, total body water, and bone mineral content. Total body density was determined by whole-body air-displacement plethysmography using the BOD POD version 1.69 (Body Composition System; Life Measurement), as described previously (29). Each participant was tested in a 1-piece swimsuit and Lycra swim cap. Same-day repeat measures of body density by the BOD POD in our laboratory had an intraclass correlation of *r* = 0.98 and SEE of 0.00365 (g/cm^3^). The room that housed the BOD POD was well ventilated between tests. Body weight was measured with an electronic scale while the subjects were in a fasting state and immediately after they had voided in the morning. Total body water was determined by isotope dilution with the use of deuterium and ^18^O-labeled water as previously described (30). Bone mineral content was determined by DXA (DPX-L; Lunar Corp) with the use of software version 1.5g (Lunar Corp).

#### RMR

Three consecutive mornings after an overnight stay in the GCRC and 12-h fast, RMR was measured immediately after awakening between 06:00 and 07:00 h. Subjects were not allowed to sleep and measurements were made in a quiet, softly lit, well-ventilated room. Temperature was maintained between 22° and 24°C. Subjects were allowed to use a cover if desired. Measurements were made supine on a comfortable bed, with the head enclosed in a plexiglass canopy. After resting for 15 min, RMR was measured for 30 min with a computerized, open-circuit, indirect calorimetry system with a ventilated canopy (Delta Trac II; Sensor Medics). The last 20 min of measurement was used for analysis. Oxygen uptake (VO_2_) and carbon dioxide production (CO_2_) were measured continuously, and values were averaged at 1-min intervals. The CV for the repeat RMR was <4%.

### Statistical analysis

Statistical analysis was performed with SPSS version 22 (SPSS, Inc.). Data are presented as means ± SDs, and statistical significance was set at *P* < 0.05. Changes in body weight/composition and RMR over time were assessed with a repeated-measures ANOVA, using Bonferroni correction for multiple comparisons. The presence of metabolic adaptation was tested by paired *t* tests, comparing measured RMR (RMRm) and predicted RMR (RMRp) at the same time points. Two equations to predict RMR were derived from baseline data of all 171 participants (participants who at least finished the weight-loss intervention and had body-composition data from 4CM available at baseline) who were part of this analysis (88 whites), aged 35.2 ± 6.3 y and with a BMI of 28.3 ± 1.3. One equation used body composition derived from the 4CM and the other from DXA.

Model 1 (derived from 4CM): RMRp (kcal/d) = 542.279 – [3.726 × age (y)] − {[114.519 × race (0 for whites, 1 for blacks] + [2.930 × FM (kg)] + [20.686 × FFM (kg)]}

(1) \begin{eqnarray*} {R^2} = {\rm{0.40}}\,;\,\,P\,\,\lt \,\,0.001 \end{eqnarray*}

Model 2 (derived from DXA): RMRp (kcal/d) = 520.571 – [2.894 × age (y)] − {[110.519 × race (0 for whites, 1 for blacks)] + [2.704 × FM (kg)] + [22.825 × FFM (kg)]}

(2) \begin{eqnarray*} {R^2} = {\rm{0.40}}\,;\,\,P\,\,\lt \,\,0.001 \end{eqnarray*}

This small *R*^2^ is due to the study design of the parent studies in which a very narrow range of BMIs and ages and only women were included.

Given that no differences were found in study outcomes between the 2 regression models, results are only given for data derived from model 1. Duration of weight loss was not a significant predictor in any of the models and only increased *R*^2^ by 2%. Moreover, its inclusion did not change the main outcomes of the analysis. For those reasons, this variable was not included in the prediction models.

Correlation analysis was performed between metabolic adaptation after weight loss, weight, FM, and FFM loss (baseline after weight loss) and weight regain at 1 and 2 y (as a % of the initial weight lost), using Pearson or Spearman correlation coefficients, as appropriate. Differences in metabolic adaptation between races were investigated by independent-samples *t* test.

## Results

Baseline characteristics of the study participants can be seen in **Table 1**. A total of 171 women (88 whites) with a mean BMI of 28.3 ± 1.3 and a mean age of 35.2 ± 6.3 y were included in the present analysis. No significant differences were seen in age, weight, height or BMI between races; however, FM (both in % and kilograms) was significantly higher in whites compared with blacks (*P* = 0.01 and *P* = 0.022, respectively).

**FIGURE 1:**
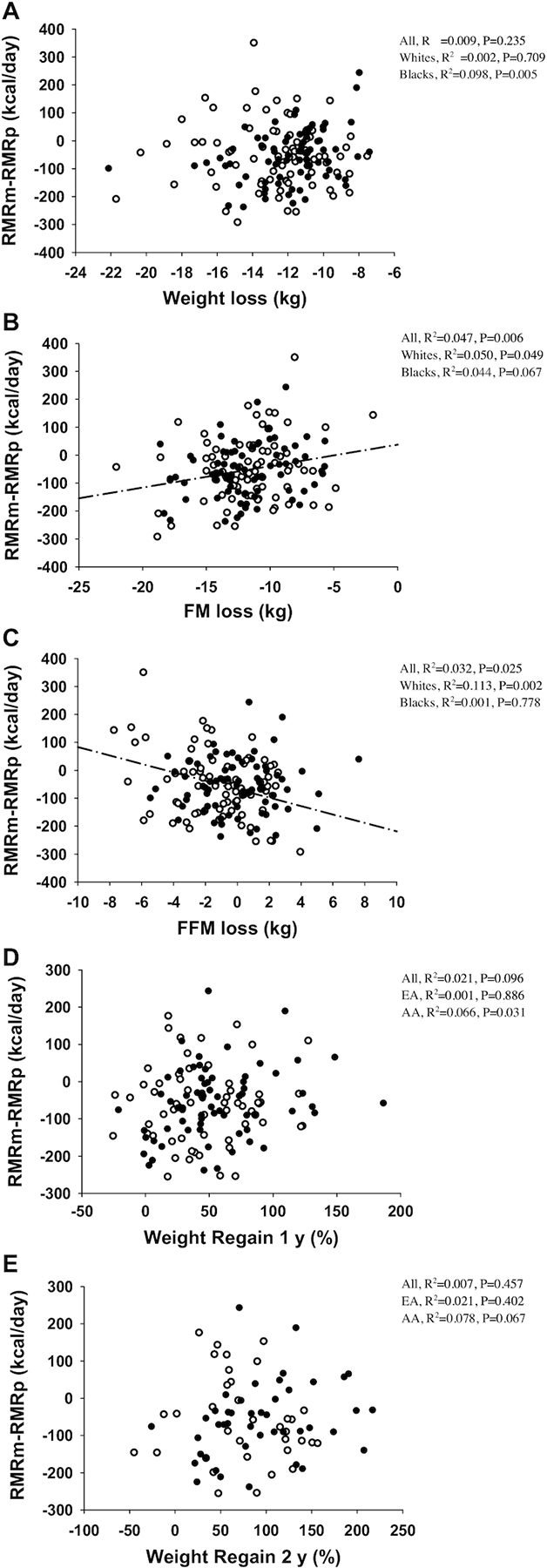
Correlation between metabolic adaptation at the level of resting metabolic rate (RMRm – RMRp) and weight loss (A), FM loss (B), and FFM loss (C), weight regain (%) at 1 y and 2 y (D, E) in all participants (*n* = 156), whites (open circles; *n* = 79), and blacks (black filled circles; *n* = 77). A negative value in the *y* axis is indicative of metabolic adaptation. A greater metabolic adaptation was associated with more weight loss in blacks; more FM loss in all women, whites, and blacks (only trend); less FFM loss in all women and whites; and less weight regain in blacks (significant at 1 y, trend at 2 y). A regression line is shown when correlations were significant in white women. FFM, fat-free mass; FM, fat mass; RMRm, resting metabolic rate measured; RMRp, resting metabolic rate predicted.

**TABLE 1:** Baseline characteristics of the study participants^1^

Characteristics	All (*n* = 171)	Whites (*n* = 88)	Blacks (*n* = 83)
Age, y	35.2 (34.3, 36.2)	35.5 (34.2, 36.9)	35.0 (33.6, 36.3)
Anthropometrics			
BMI, kg/m^2^	28.4 (28.2, 28.6	28.5 (28.2, 28.7)	28.4 (28.0, 28.7)
Weight, kg	77.6 (76.5, 78.7)	78.5 (76.9, 80.2)	76.6 (75.2, 78.1)
Height, cm	165.2 (164.2, 166.2)	166.0 (164.6, 167.4)	164.4 (162.9, 165.8)
Fat mass kg	32. 3 (31.5, 33.1)	33.3 (32.1, 34.5)**	31.2 (30.3, 32.2)**
Fat mass %	41.5 (40.9, 42.1)	42.2 (41.2, 43.2)*	40.7 (39.8, 41.6)*
Fat-free mass, kg	45.3 (44.7, 46.0)	45.2 (44.3, 46.2)	45.4 (44.5, 46.3)
Fat-free mass, %	58.5 (57.9, 59.2)	57.8 (56.8, 58.8)	59.3 (58.5, 60.2)

1 Values are means (95% CIs). Differences between races were assessed with independent-samples *t* tests. ^*,**^Different between whites and blacks: *P = 0.022, **P = 0.01.

Average weight loss was −12.2 ± 2.6 kg (−15.7% ± 2.9%), achieved over an average of 153 ± 53 d. Whites lost significantly more weight compared blacks in absolute terms (−12.7 ± 2.8 vs −11.8 ± 2.4 kg; *P* = 0.045), but not when weight loss was expressed as a percentage of the initial body weight (−16.1% ± 3.1% vs −15.3% ± 2.7%; *P* = 0.110). Weight regain was, on average, 51.5% ± 37.6% at 1 y (*n* = 131), with no statistically significant differences between whites and blacks (47.2% ± 36.2% vs 55.2% ± 38.6%; *P* = 0.223) and 88.8% ± 54.4% at 2-y follow-up (*n* = 79), with no statistically significant differences between whites and blacks (75.9% ± 49.1% vs 95.4% ± 56.8%; *P* = 0.115).

RMRm was significantly lower than RMRp after weight loss in all participants (*n* = 156) [whites (*n* = 79) and blacks (*n* = 77): 1291 ± 136 vs 1345 ± 103, 1338 ± 135 vs 1389 ± 93, and 1243 ± 121 vs 1301 ± 93 kcal/d, respectively; *P* < 0.001 for all], resulting in a metabolic adaptation of −54 ± 105 kcal/d and −52 ± 115 and −57 ± 94 kcal/d, respectively, with no significant differences between whites and blacks. Metabolic adaptation after weight loss was not correlated with weight loss or weight regain at 1 or 2 y of follow-up but was positively correlated with FM loss and negatively correlated with FFM loss, in all participants and whites. In blacks, metabolic adaptation was positively correlated with weight loss and FM loss (only trend, *P* = 0.067) and negatively correlated with weight regain at both 1 and 2 y (only trend, *P* = 0.067) (**Table 2** and **Figure 1**).

**TABLE 2:** Correlation analysis between metabolic adaptation, weight loss, and weight regain at 1 and 2 y^1^

	*r*	*P*	*n*
Weight loss			
All	0.096	0.235	156
Whites	−0.043	0.709	79
Blacks	0.314	0.005	77
FM loss			
All	0.218	0.006	156
Whites	0.223	0.049	79
Blacks	0.210	0.067	80
FFM loss			
All	−0.180	0.025	156
Whites	−0.336	0.002	79
Blacks	−0.033	0.778	77
Weight regain at 1 y			
All	0.146	0.096	131
Whites	0.019	0.886	61
Blacks	0.258	0.031	70
Weight regain at 2 y			
All	0.085	0.457	79
Whites	−0.146	0.402	35
Blacks	0.278	0.067	44

1 *r* indicates correlation coefficients from Spearmen correlation.

Anthropometrics, RMRm, and RMRp, as well as metabolic adaptation (RMRm – RMRp), over time in those women with data at all points (*n* = 46) can be seen in **Tables 3** and **4**. On average, women in this subsample had an average weight loss of 12 ± 3 kg and a weight regain of 52% ± 36% and 83% ± 52% at 1- and 2-y follow-up, respectively. There was a significant metabolic adaptation after weight loss in all participants and blacks [−43 ± 119 kcal/d (*P* = 0.019) and −50 ± 113 kcal/d (*P* = 0.047), respectively] but not in whites (−35 ± 126 kcal/d; *P* = 0.187). No metabolic adaptation was seen at 1- and 2-y follow-up in all participants, whites or blacks.

**TABLE 3:** Anthropometrics and RMR data over time^1^

					*P* value^2^		
	Baseline (*n* = 46/23)	After WL (*n* = 46/23)	1 y (*n* = 46/23)	2 y (*n* = 46/23)	Baseline vs WL	Baseline vs 1 y	Baseline vs 2 y
Weight, kg							
All	78.0 ± 7.0	65.7 ± 6.1	71.7 ± 8.2	75.1 ± 9.3	<0.001	<0.001	0.026
Whites	80.1 ± 7.9	66.7 ± 7.0	73.3 ± 8.9	75.4 ± 9.5	<0.001	<0.001	0.023
Blacks	76.0 ± 6.3	64.7 ± 5.6	70.3 ± 7.4	74.8 ± 9.2	<0.001	<0.001	1.000
FM, kg							
All	31.9 ± 5.2	20.4 ± 4.1	25.8 ± 6.0	28.9 ± 7.2	<0.001	<0.001	0.009
Whites	33.7 ± 6.2	22.2 ± 4.3	27.7 ± 6.4	30.1 ± 7.5	<0.001	0.001	0.171
Blacks	30.2 ± 3.4	18.7 ± 3.2	24.0 ± 5.2	27.7 ± 6.8	<0.001	<0.001	0.128
FFM, kg							
All	46.1 ± 4.1	45.3 ± 4.3	46.0 ± 4.2	46.3 ± 4.1	0.257	1.000	0.999
Whites	46.4 ± 4.3	44.5 ± 4.1	45.6 ± 4.1	45.3 ± 4.0	0.034	0.820	0.835
Blacks	45.8 ± 3.9	46.0 ± 4.4	46.3 ± 4.3	47.2 ± 4.1	0.999	0.968	0.005
RMRm, kcal/d							
All	1392 ± 137	1307 ± 152	1358 ± 166	1369 ± 193	<0.001	0.465	0.999
Whites	1452 ± 133	1353 ± 145	1401 ± 164	1422 ± 200	0.024	0.520	0.999
Blacks	1334 ± 117	1261 ± 149	1316 ± 159	1316 ± 174	0.040	0.999	0.999
RMRp, kcal/d							
All	1398 ± 109	1348 ± 104	1374 ± 109	1386 ± 106	<0.001	0.001	0.604
Whites	1461 ± 93	1388 ± 97	1424 ± 95	1422 ± 98	<0.001	0.002	0.029
Blacks	1338 ± 87	1309 ± 97	1326 ± 10	1351 ± 103	0.030	0.844	0.812

1 Values are means ± SDs. n in all/whites. Changes over time were analyzed with a repeated measures ANOVA. FFM, fat-free mass; FM, fat mass; RMR, resting metabolic rate; RMRm, RMR measured; RMRp, RMR predicted; WL, weight loss.

2 *P* values for post hoc comparisons between time points after Bonferroni adjustment.

**TABLE 4:** Metabolic adaptation (RMRm – RMRp) over time^1^

					*P* value			
	Baseline (*n* = 46/23)	After WL (*n* = 46/23)	1 y (*n* = 46/23)	2 y (*n* = 46/23)	Baseline	After WL	1 y	2 y
RMRm–RMRp, kcal/d								
All	−7 ± 102	−*43 ± 119*	−18 ± 134	−19 ± 166	0.643	*0.019*	0.380	0.438
Whites	−9 ± 104	−35 ± 126	−23 ± 134	0.2 ± 198	0.689	0.187	0.416	0.997
Blacks	−5 ± 103	−*50 ± 113*	−12 ± 137	−38 ± 127	0.583	*0.04^b^*	0.681	0.161

1 Values are means ± SDs. n in all/whites. RMR, resting metabolic rate; RMRm, RMR measured; RMRp, RMR predicted; WL, weight loss.

In a subset of women with data at all time points (*n* = 48), metabolic adaptation was present after weight loss, but not at 1- and 2-y follow-up [−64 ± 106 kcal/d (*P* < 0.001), −26 ± 132 (*P* = 0.076), and −20 ± 155 kcal/d (*P* = 0.358), respectively].

## Discussion

The present findings represent the first longitudinal study examining metabolic adaptation, with measurements taken under conditions of weight stability. After a 12-kg (16%) weight loss, an RMR metabolic adaptation of ∼50–60 kcal/d below predicted levels was found regardless of race. However, metabolic adaptation was not sustained at 1 or 2 y of follow-up (average weight regain: 52% and 83%, respectively).

Even though we did not confirm our hypothesis that metabolic adaptation would be absent under conditions of weight stability, metabolic adaptation was minor (50 kcal/d; 3–4%) after weight loss. Two reasons may explain this phenomenon. The first is that 4 wk of weight stabilization may not be enough for metabolic adaptation to disappear. The second, and likely more plausible, explanation is that our participants, despite being weight stable, were probably in negative EB when measurements were performed after weight loss. Weight loss in the present study was induced by an 800-kcal/d diet, which, due to its highly restrictive nature (112–116 g carbohydrate/d), was most likely a ketogenic diet. Even though measures of ketosis are not available, studies using similar diets have reported participants to be ketotic (31, 32). The physiological state of ketosis is accompanied by partial glycogen depletion and, with it, water loss, while refeeding is followed by glycogen replenishment and, with it, increased water content. It has been estimated that glycogen stores are, on average, 400–500 g (33, 34), with 3–4 g of water bound to each gram of glycogen (33). This means that an increase in body weight of ∼1 kg, due to increased water content, should be expected when participants come out of ketosis.

The aspects discussed previously are of paramount relevance as they are likely to explain the discrepancy in the literature regarding the existence or not of metabolic adaptation. All the studies reporting no metabolic adaptation at the level of RMR are studies where weight-stable, reduced-obesity individuals were compared with never-obese BMI-matched controls (2, 4, 18, 35) or against a regression model (20). In contrast, longitudinal studies tend to find metabolic adaptation (1, 9, 36), likely because measurements are taken under negative EB. For example, in the landmark paper by Leibel and colleagues (1), even though participants were weight stable for 2 wk, they were, most likely, in negative EB as a 800-kcal/d ketogenic diet was used to induce weight loss (as in the present study). Results from the “Biggest Loser” study suffer from the same problem, as participants were clearly in negative EB at the end of the 30-wk competition and, even at 6-y follow up, there was a very large interindividual variation in weight stability, with some participants gaining up to 3 kg and some losing up to 3 kg over the 2 wk preceding RMR measurement (9, 36).

If metabolic adaptation was part of a compensatory response that tries to bring body weight back to its original state and, therefore, a driver of weight regain, then it would be expected that a larger metabolic adaptation was associated with more weight regain long term. That is not the case, either in the present analysis or in the available literature (9). In fact, the evidence suggests metabolic adaptation to be a reflection of the magnitude of weight loss: the larger the weight loss, the larger the metabolic adaptation (9, 10, 36). This pattern was observed in the present analysis among blacks, where a larger metabolic adaptation was associated with greater weight loss and less weight regain. The reason for this race difference is not clear, but differences in the composition of FFM between whites and blacks may be a possible source of confounding. The fact that a greater metabolic adaptation is, in fact, associated with less weight regain, as shown in blacks in the present analysis and also in the Biggest Loser study at 6-y follow up, is likely to reflect the fact that the larger the initial weight loss, the less the weight regain long term (*r* = −0.270, *P* = 0.001, *n* = 143, and *r* = −0.283, *P* = 0.008, *n* = 87 at 1- and 2-y follow-up, respectively, in the present analysis). This adds to the previously proposed idea that metabolic adaptation is a mere reflection of the magnitude of weight loss, both in the short and long term.

In line with the evidence previously discussed for RMR, the existence of metabolic adaptation at the level of nonresting EE after weight loss (due supposedly to increased exercise efficiency) is also likely to be modulated by the EB status of the participants when measurements are taken. As such, no metabolic adaptation was found in nonresting EE (3, 37–39) following a 10- to 12-kg weight loss in overweight premenopausal women when measurements were done in controlled conditions of weight stability. Moreover, to our knowledge, no study has ever reported increased exercise efficiency with weight loss to be associated with long-term weight regain. In fact, improved locomotion economy/efficiency may actually reduce the risk of weight regain, as several studies have shown that exercise training–induced increases in exercise economy are associated with increased ease of locomotion (40–43), which, in turn, is associated with increased participation in free-living physical activity and reduced weight regain (44–48).

Therefore, the concept of metabolic adaptation as a major driver of weight regain should be put to rest. Despite relapse continuing to be the norm in obesity management (49–51), it is time for the scientific community to accept that weight regain in not an inevitability driven by strong metabolic adaptation at the level of EE and to move on. If obesity is treated as what it is—a “chronic relapsing disease” (52)—with patients receiving long-term support, aiming at keeping a healthy lifestyle, then relapse can be minimized or even prevented (53).

Our study has both strengths and limitations. The main strength is its design, with data collected under conditions of weight stability at all time points. This is important for 2 reasons: first, because it quality-assured that our prediction model is based on baseline data; second, because it allowed the measurement of metabolic adaptation under conditions of weight stability. Moreover, gold-standard procedures were used both for the measurements of RMR (after a 4-d GCRC inpatient stay and an overnight sleep, under controlled condition of feeding and physical activity) and body composition (4CM). However, this study also suffers from some limitations. First, it included a very homogenous sample of premenopausal (20–41 y) overweight women. This prevents the generalizability of our results to men, other BMI groups, and older subjects. Second, this also explains why our regression model had an *R*^2^ of only 40% (i.e., a truncated range for both BMI and age and only women). Third, there was a relative high dropout rate at 1- and 2-y follow-up and it is possible that those who dropped out were significantly different from those who completed the intervention. It has been shown that participants who drop out during weight-loss studies tend to have a higher baseline BMI and a younger age (54) and experience less weight loss during the active weight-loss phase of the program (55). Finally, our participants were likely in negative EB after weight loss, despite being maintained under conditions of weight stability for 4 wk, meaning that our estimates of metabolic adaptation are probably exaggerated.

In conclusion, metabolic adaptation at the level of RMR is minor when measurements are taken under weight stability and is not sustained in the long term with weight regain. More importantly, metabolic adaptation does not predict relapse in the long term. Further research should explore alternative mechanistic pathways that can explain weight regain, which includes both physiological and behavioral aspects.

## Supplementary Material

nqaa086_Supplemental_File

## Notes

Supported by grants National Institutes of Health grants R01 DK049779, P30 DK56336, P60 DK079626, and UL 1RR025777. CM was supported by a sabbatical grant from the Liaison Committee for Education, Research and Innovation in Central Norway and the Norwegian University of Science and Technology (NTNU).

Supplemental Figure 1 is available from the “Supplementary data” link in the online posting of the article and from the same link in the online table of contents at https://academic.oup.com/ajcn/.

Data described in the manuscript will not be made available because that would violate Institutional Review Board and Health Insurance Portability and Accountability Act rules.

Abbreviations used: EB, energy balance; EE, energy expenditure; FM, fat mass; FFM, fat-free mass; GCRC, General Clinical Research Center; RMR, resting metabolic rate; RMRm, resting metabolic rate measured; RMRp, resting metabolic rate predicted; TEE, total energy expenditure; UAB, University of Alabama at Birmingham; 4CM, 4-compartment model.
