# Supplementary material for: Metabolic adaptation is not a major barrier to weight-loss maintenance
Source: Am J Clin Nutr. 2020 May 9;112(3):558–65. doi: 10.1093/ajcn/nqaa086 (PMC7458773; doi:10.1093/ajcn/nqaa086)
Supplement: nqaa086_Supplemental_File [file nqaa086_supplemental_file.pdf]

On-line Supplementary Material

Martins et al. Metabolic adaptation is not a major barrier to weight loss maintenance

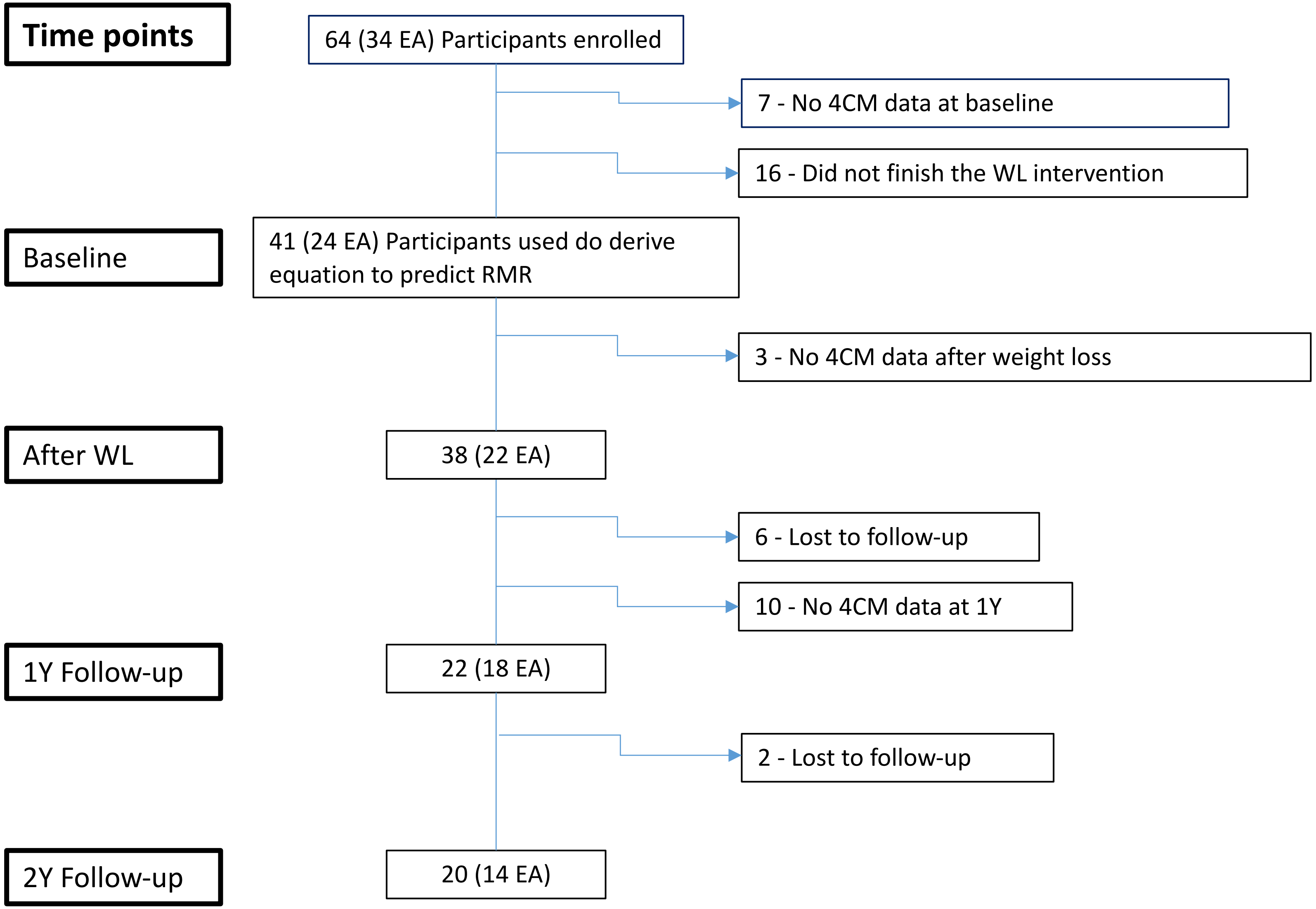

Supplementary Figure 1A. Flowchart ROMEO study

EA: European American; 4CM: 4 Compartment Model; WL: weight loss; RMR: resting metabolic rate; Y: year

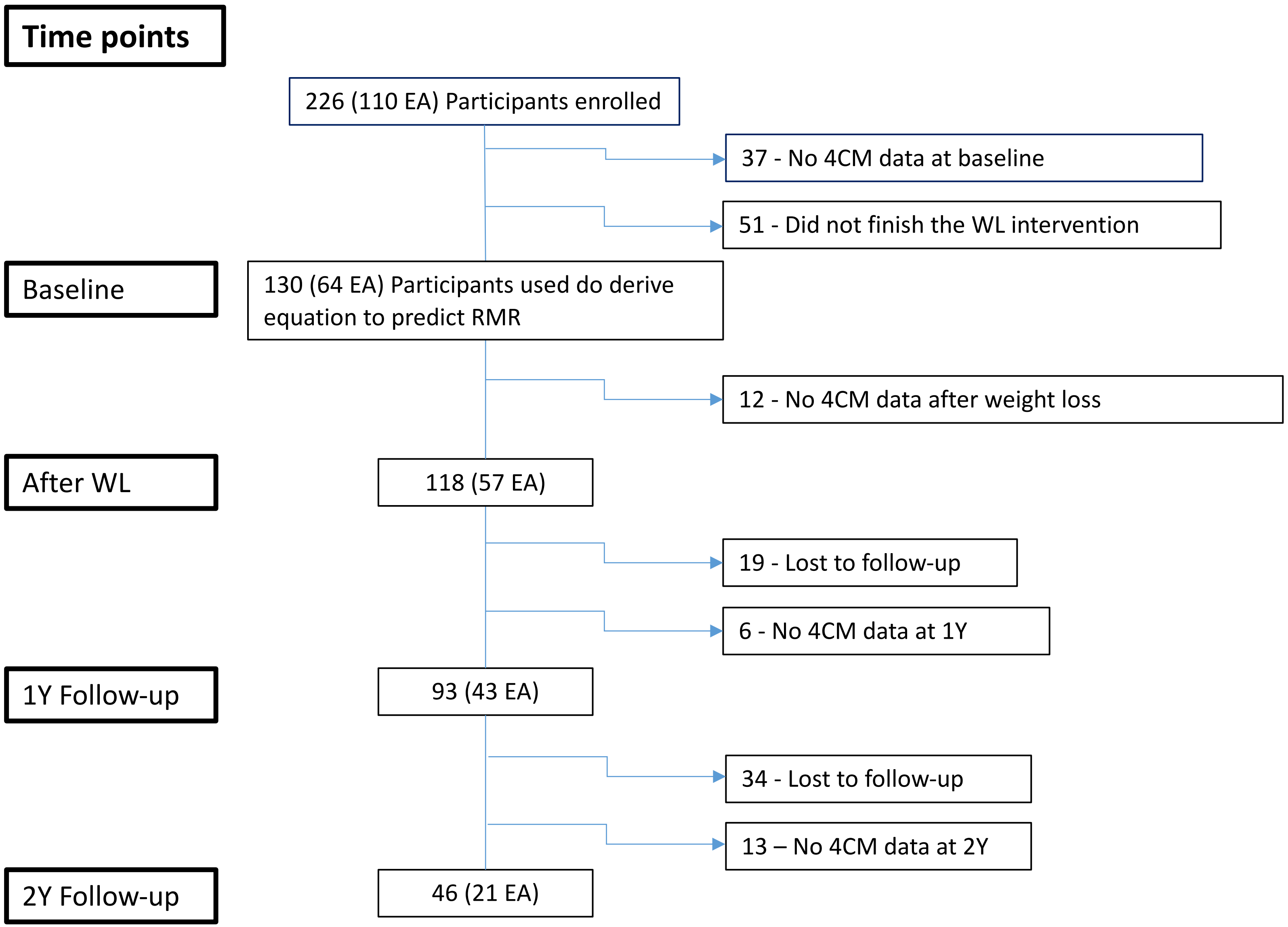

Supplementary Figure 1B. Flowchart JULIET study

EA: European American; 4CM: 4 Compartment Model; WL: weight loss; RMR: resting metabolic rate; Y: year
